# Physical function, disease activity, and health-related quality-of-life outcomes after 3 years of adalimumab treatment in patients with ankylosing spondylitis

**DOI:** 10.1186/ar2790

**Published:** 2009-08-17

**Authors:** Désirée M van der Heijde, Dennis A Revicki, Katherine L Gooch, Robert L Wong, Hartmut Kupper, Neesha Harnam, Chris Thompson, Joachim Sieper

**Affiliations:** 1 Department of Rheumatology, Leiden University Medical Center, Leiden, PO Box 9600, 2300 RC Leiden, The Netherlands; 2 Center for Health Outcomes Research, United BioSource Corporation, 7101 Wisconsin Avenue, Suite 600, Bethesda, Maryland, 20814, USA; 3 Global Health Economics and Outcomes Research, Abbott Laboratories, 100 Abbott Park Road, Abbott Park, Illinois, 60064, USA; 4 Formerly Abbott Immunology, Abbott Laboratories, 300 Interpace Parkway, Parsippany, New Jersey, 07054, USA; 5 Abbott GmbH & Co. KG, Knollstrasse 50, Ludwigshafen, 67061, Germany; 6 Medical Department I, Rheumatology, Charité, Campus Benjamin Franklin, and German Rheumatism Research Center, Hindenburgdamm 30, Berlin, 12200, Germany

## Abstract

**Introduction:**

We evaluated the three-year impact of adalimumab on patient-reported physical function and health-related quality-of-life (HRQOL) outcomes in patients with active ankylosing spondylitis (AS).

**Methods:**

The Adalimumab Trial Evaluating Long-Term Efficacy and Safety in AS (ATLAS) is an ongoing five-year study that included an initial 24-week, randomized, placebo-controlled, double-blind period, followed by open-label extension treatment with adalimumab. Clinical and HRQOL data collected for up to three years from ATLAS were used for these analyses. Patients were randomized to receive adalimumab 40 mg or placebo by subcutaneous injection every other week. Physical function was assessed by the Bath AS Functional Index (BASFI), as well as by the Short Form 36 (SF-36) Health Survey Physical Component Summary (PCS) and Physical Function subscale scores. HRQOL was assessed using the AS Quality of Life (ASQOL) questionnaire. Disease activity was assessed by the Bath AS Disease Activity Index (BASDAI).

**Results:**

Of 315 patients enrolled in ATLAS, 288 (91%) participated in an open-label adalimumab treatment extension and 82% provided three-year outcome data. During the 24-week double-blind phase, adalimumab-treated patients experienced significant improvement compared with placebo-treated patients in the BASDAI (*P *< 0.001), BASFI (*P *< 0.001), ASQOL (*P *< 0.001), and both the SF-36 PCS (*P *< 0.001) and Physical Function subscale (*P *< 0.001) scores, but not the SF-36 Mental Component Summary score (*P *= 0.181) and Mental Health subscale scores (*P *= 0.551). Mean changes from baseline through three years of adalimumab treatment were statistically significant for the BASDAI (change score: -3.9, *P *< 0.001), BASFI (change score: -29.6, *P *< 0.001), SF-36 PCS (change score: 11.6, *P *< 0.001), and Physical Function (change score: 23.3, *P *< 0.001). Comparable results were observed for the other SF-36 scores and for the ASQOL (all *P *< 0.001).

**Conclusions:**

Adalimumab significantly improved disease activity, patient-reported physical function, and HRQOL. These benefits were maintained over three years of treatment in patients with AS.

**Trial registration:**

ClinicalTrials.gov NCT00085644.

## Introduction

Ankylosing spondylitis (AS) is a chronic, inflammatory, systemic, rheumatic disease that primarily affects the axial skeleton, peripheral joints, and entheses [1]. Symptoms of AS include pain, joint stiffness, and the loss of spinal mobility. These clinical symptoms and the subsequent disease progression result in substantial functional limitations and impairment of health-related quality of life (HRQOL) [2-5]. Patient-reported outcome (PRO) measures have been used to provide information on the effectiveness of treatment on symptoms, functioning, and well-being outcomes. PRO measures are necessary tools, given the impact of AS on HRQOL domains, especially pain, physical function, fatigue, and psychological well-being.

Currently, two PRO instruments have been employed in the evaluation of HRQOL in AS. These are the Short Form 36 (SF-36) Health Survey, a generic measure of health status [6], and the AS Quality of Life Questionnaire (ASQOL) [7]. Many AS studies have used the SF-36 [8-19], whereas use of the ASQOL has been somewhat limited [9,16,20,21]. These measures have demonstrated HRQOL impairment and loss of physical functioning for patients with AS, compared with the general population. Using median SF-36 summary scores, van der Heijde and colleagues [15] demonstrated that baseline values of SF-36 Physical Component Summary (PCS) scores in patients with AS were less than the scores for the general populations of the US and Europe, while SF-36 Mental Component Summary (MCS) scores were comparable with those general populations. At least one other study reported statistically lesser baseline SF-36 scores for all eight SF-36 domains, especially those pertaining to physical function, for patients with AS compared with the US general population [22].

The availability of new imaging techniques, therapies, and treatments over the past several years has changed the management of AS [23]. Previously, treatment options for AS were limited to nonsteroidal anti-inflammatory drugs (NSAIDs) and physiotherapy. With the availability of TNF antagonists (infliximab, adalimumab, and etanercept), AS patients have experienced improvements in the signs and symptoms of their disease. In clinical trials of TNF antagonists, improvements in clinical symptoms of AS have been sustained, lasting up to three years [11,24], although treatment discontinuation has been associated with relapse [24,25]. TNF antagonists in the treatment of AS have proven efficacy for patients experiencing treatment-resistant forms of AS [26,27], for patients seeking to reduce the use of NSAIDs and analgesics [28], and for patients seeking effective low-dosage [29,30] and less-frequent treatments [12,31].

Adalimumab, a fully human monoclonal antibody targeted against TNF, has demonstrated short-term improvement in clinical signs and symptoms, physical function, and HRQOL in patients with active AS [16,32]. Maintenance of improvement in clinical signs and symptoms (without new safety issues) has been demonstrated with adalimumab in patients who continued in an open-label extension trial [33]. However, in light of the substantial impairment of physical function and HRQOL observed in patients with active AS, additional long-term data are needed to determine the maintenance of benefit on both physical function and quality of life. Sustained improvements in HRQOL have been demonstrated through 72 weeks of etanercept therapy based on the SF-36 PCS and the EQ-5D [19] and through two years of infliximab therapy based on the SF-36 PCS [18], but neither study assessed HRQOL using the ASQOL, an AS-specific questionnaire. The primary objective of our analyses was to demonstrate the effectiveness of adalimumab after three years of treatment in improving and sustaining patient-reported physical functioning and HRQOL.

## Materials and methods

### Patients and study design

The Adalimumab Trial Evaluating Long-Term Efficacy and Safety in AS (ATLAS) was a multicenter, randomized, double-blind, placebo-controlled, phase III study designed to demonstrate the safety and efficacy of adalimumab in patients with active AS. Complete study information for ATLAS has been previously published [16,17,32]. Patients who were at least 18 years of age were recruited from 43 sites (21 in the US and 22 in Europe). Eligibility criteria included a diagnosis of AS according to the modified New York criteria [34] and an inadequate response or intolerance to at least one NSAID. Patients for whom one or more disease-modifying antirheumatic drug had failed were also allowed to participate. Patients were randomized in a 2:1 ratio to receive either adalimumab 40 mg or matching placebo subcutaneously every other week for 24 weeks (Abbott Laboratories, Abbott Park, IL, USA). Participants who did not achieve at least a 20% response according to the Assessment of SpondyloArthritis International Society criteria for improvement at weeks 12, 16, or 20 were eligible to receive open-label treatment with adalimumab 40 mg every other week (early escape therapy). After week 24, patients were eligible to continue adalimumab treatment in an open-label extension study for up to five years.

Clinical and HRQOL data from both the 24-week double-blind period and the open-label extension of the ATLAS clinical trial were used for the data analyses described in this report. Relevant institutional review boards at participating clinical centers approved the protocol, and all patients provided voluntary written informed consent.

### Measures

Three PRO instruments – the Bath AS Functional Index (BASFI) [35], the SF-36 Health Survey [6], and the ASQOL [7] – were used in the long-term follow-up of adalimumab treatment. The Bath AS Disease Activity Index (BASDAI) was included as a measure of disease activity [36].

#### BASFI

The BASFI consists of 10 questions designed to determine the degree of functional limitation in patients with AS. Each question is answered using a 10-cm visual analogue scale (VAS), with a recall period of the past week. The mean of the 10 scales gives the BASFI score – a value between 0 and 10, with a lower score indicating less functional limitation. The BASFI has been found to be reliable and sensitive to changes in AS [35,37]. Pavy and colleagues [38] suggested a BASFI change of 17.5% is the minimum clinically important difference for AS patients. For our study, however, we employed a 21% or higher BASFI change as clinically meaningful, [39] because it is more conservative and more consistent with Assessment in AS International Group Criteria for 20% improvement (ASAS20) response. The ASAS20 response includes assessment of BASFI scores [40], and was the primary endpoint of ATLAS.

#### SF-36

SF-36 is a generic health status instrument and consists of eight domains: Physical Function, Bodily Pain, Role Limitations–Physical (Role-Physical), General Health, Vitality, Social Function, Role Limitations–Emotional (Role-Emotional), and Mental Health. The recall period is four weeks, with greater scores reflecting better health status. A 5- to 10-point change in domain scores is considered clinically meaningful for patients with rheumatoid arthritis [41]. SF-36 also contains two summary scores (PCS and MCS) for which a 2.5 to 3.0-point change in summary scores is considered clinically meaningful for patients with rheumatoid arthritis [41]. Previous studies in patients with AS have used three or more points to determine a clinically meaningful change [16].

#### ASQOL

The ASQOL is a disease-specific instrument designed to measure HRQOL in patients with AS [7]. This questionnaire was developed according to a needs-based model, which postulates that life gains its quality from the ability of individuals to satisfy their needs. The final instrument contains 18 yes/no items on the impact of AS 'at this moment'. The total score ranges from 0 to 18, with lesser scores representing better AS-specific quality of life. Recent research has suggested that the ASQOL is a psychometrically sound and responsive measure of disease-specific quality of life in patients with AS and differences of one to two points are clinically relevant [7,42,43].

#### BASDAI

The BASDAI is a patient-reported measure of AS disease activity. This index uses six 10-cm horizontal VASs to measure the severity of fatigue, spinal and peripheral joint pain, localized tenderness, and morning stiffness in patients with AS. The final BASDAI score has a range of 0 to 10; a lesser number represents less severe disease activity [36].

### Statistical analyses

The statistical analyses used data from the ATLAS clinical trial. A two-tailed *P *value of 0.05 was used to judge statistical significance. There was no adjustment for multiple statistical comparisons; however, interpretation of significant findings took the number of statistical tests into account. The sample included all patients randomized in the ATLAS trial who had a baseline PRO assessment and at least one follow-up PRO assessment for the analyses of the 24-week double-blind study period. Furthermore, we included only those patients who had a week-24 BASFI assessment and at least one more post-24-week assessment. For the long-term extension period, the analyses are based on actual study visit data (ie, weeks from baseline of the ATLAS trial), not on duration of exposure to adalimumab.

We compared baseline demographic variables, selected clinical characteristics, and PRO measures between the randomized, double-blind clinical trial patient sample and the open-label extension patient sample to evaluate the extent of sample bias. We used chi-square tests for categorical data and paired Student *t-*tests for independent groups for continuous variables.

We compared baseline to week-24 changes in mean BASDAI, BASFI, SF-36 summary scales and subscales, and ASQOL scores between placebo-treated and adalimumab-treated groups during the double-blind treatment period using analysis of covariance (ANCOVA) models. ANCOVA models included baseline PRO score and treatment group. Last-observation-carried-forward (LOCF) procedures were used to account for PRO scores after patients went into early escape therapy or who discontinued from the study. If patients started on early escape treatment before the 24-week endpoint, their last complete pre-escape therapy PRO scores were used as the endpoint score for the LOCF.

For selected PRO measures (SF-36 PCS, BASFI, and ASQOL), chi-square tests were used to compare the percentage of responders, based on changes from baseline to week 24, between the placebo-treated and adalimumab-treated groups. The minimum clinically important difference (MCID) from baseline for each PRO was defined as follows: SF-36 PCS responder, three or more point reduction; BASFI responder, 21% or more reduction; and ASQOL responder, 1.8 or more point reduction. The percentages of responders for these PRO measures also were evaluated from baseline to each open-label extension follow-up visit, using these same MCID definitions.

Long-term follow-up analyses for the open-label extension period were completed using observed data. For the BASDAI, BASFI, SF-36 summary and subscale, and ASQOL scores, we calculated the change in scores from baseline to each follow-up visit and used paired Student *t-*tests to assess significance of the observed changes. Changes from week 24 to each long-term follow-up visit also were calculated for the BASDAI, BASFI, and SF-36 summary and ASQOL scores. For the BADAI, BASFI, and PCS scores, we also examined mean changes during the open-label extension (i.e., from week 24 on), based on LOCF analyses.

Effect sizes were calculated for changes in PRO measures as (pretreatment mean – posttreatment mean)/pretreatment standard deviation [44]. Effect sizes were classified as small (0.20), moderate (0.50), or large (≥ 0.80) [45].

## Results

### Patients

A total of 315 patients with active AS participated in the ATLAS study; 208 were randomized to receive adalimumab and 107 to receive placebo. Most patients were white (95.6%) and male (74.9%). The average age was 42.2 years, and mean disease duration was 10.9 years. A total of 288 patients (91.4%) entered the open-label extension phase of the study. Of these 288, 236 (81.9%) had data for three years beyond their baseline visits in the ATLAS study. Of the 52 patients who enrolled in the open-label extension but did not complete three years, 17 discontinued because of adverse events.

### Baseline assessments

Baseline demographic variables and mean PRO measures were comparable between the 24-week, randomized, double-blind clinical trial sample and those who participated in the open-label extension study (Table 1).

**Table 1:** Baseline demographic and clinical characteristics of patients with AS: comparison of patients entering double-blind and open-label extension study periods

	Week 24 of ATLAS (n = 315)	Open-label extension (n = 288)	*P value*^a^
Age, years	42.2 ± 11.57	42.4 ± 11.65	0.848
Male, n (%)	236 (74.9)	219 (76.0)	0.777
White, n (%)	301 (95.6)	276 (95.8)	1.000
Disease duration, years	10.9 ± 9.47	10.9 ± 9.43	0.953
BASDAI score, 0–10 cm	6.3 ± 1.69	6.3 ± 1.70	0.997
BASFI score, 0–10 cm	5.4 ± 2.21	5.4 ± 2.19	0.900
SF-36 PCS, 0–50	32.5 ± 7.98	32.4 ± 8.00	0.846
SF-36 MCS, 0–50	43.7 ± 11.57	44.0 ± 11.48	0.804
SF-36 domain scales			
Physical Function	47.1 ± 22.08	46.8 ± 22.49	0.893
Role–Physical	20.3 ± 30.02	20.9 ± 30.25	0.808
Bodily Pain	55.9 ± 25.06	55.9 ± 25.08	0.979
General Health	42.6 ± 19.82	42.6 ± 20.06	0.997
Vitality	31.1 ± 16.12	30.9 ± 16.12	0.875
Social Function	33.1 ± 17.49	32.9 ± 17.30	0.895
Role–Emotional	54.4 ± 42.98	56.1 ± 42.90	0.641
Mental Health	61.7 ± 19.19	61.8 ± 19.17	0.952
ASQOL, 0–18	10.3 ± 4.29	10.3 ± 4.29	0.945

Data are mean ± standard deviation unless otherwise noted.

^a^*P*-values for comparing means based on two-sample paired Student *t*-tests; *P*-values for comparing percentages based on Fisher's exact tests.

AS = ankylosing spondylitis; ASQOL = AS Quality of Life Questionnaire; ATLAS = Adalimumab Trial Evaluating Long-Term Efficacy and Safety in AS; BASDAI = Bath AS Disease Activity Index; BASFI = Bath AS Functional Index; HRQOL = health-related quality-of-life; MCS = Mental Component Summary; PCS = Physical Component Summary; SF-36 = Short Form-36 Health Survey.

#### Baseline to week-24 double-blind results

There were statistically significant differences between the placebo-treated and adalimumab-treated groups for baseline to week-24 changes in the PRO measures (*P *= 0.015 to *P *< 0.001), except for the SF-36 MCS (*P *= 0.181) and Mental Health subscale scores (*P *= 0.551; Table 2). For all PRO endpoints, the adalimumab group reported greater improvements.

**Table 2:** Mean changes in PRO measures from baseline to week 24 of the ATLAS study

PRO measure	Placebo (n = 107)	Adalimumab (n = 208)	Overall *F *value^a^	*P *value^c^
BASDAI, 0–10 cm	-1.0 ± 0.23	-2.8 ± 0.17	30.30^b^	< 0.001
BASFI, 0–10 cm	-0.5 ± 0.19	-2.0 ± 0.14	23.14^b^	< 0.001
SF-36 PCS, 0–50	2.1 ± 0.84	7.3 ± 0.59	24.56^b^	< 0.001
SF-36 MCS, 0–50	2.1 ± 0.95	3.7 ± 0.67	42.05^b^	0.181
Physical Function	4.1 ± 2.00	13.3 ± 1.42	19.32^b^	< 0.001
Role–Physical	10.0 ± 3.51	27.5 ± 2.51	23.00^b^	< 0.001
Bodily Pain	7.0 ± 2.03	20.7 ± 1.45	24.39^b^	< 0.001
General Health	1.5 ± 1.55	8.7 ± 1.11	21.89^b^	< 0.001
Vitality	5.8 ± 1.86	14.6 ± 1.33	21.33^b^	< 0.001
Social Function	5.0 ± 2.11	12.6 ± 1.51	34.86^b^	0.003
Role–Emotional	5.0 ± 3.55	15.6 ± 2.51	77.42^b^	0.015
Mental Health	4.8 ± 1.54	5.9 ± 1.11	30.43^b^	0.551
ASQOL, 0–18	-1.1 ± 0.40	-3.5 ± 0.28	17.28^b^	< 0.001

Data are least-square mean ± standard error.

^a^ANCOVA model controlling for baseline score of patient was performed.

^b^*P *< 0.001 based on ANCOVA.

^c^*P-*values based on paired Student *t-*tests for comparison of means between placebo-treated and adalimumab-treated patients.

ANCOVA = analysis of covariance; ASQOL = AS Quality of Life Questionnaire; ATLAS = Adalimumab Trial Evaluating Long-Term Efficacy and Safety in AS; BASDAI = Bath AS Disease Activity Index; BASFI = Bath AS Functional Index; MCS = Mental Component Summary; PCS = Physical Component Summary; PRO = patient-reported outcome; SF-36 = Short Form-36 Health Survey.

Similar differences were observed between placebo-treated and adalimumab-treated patients for the BASFI and ASQOL measures. For the SF-36 subscale and MCS scores, all differences were statistically significant (*P *< 0.05), except for the SF-36 MCS (*P *= 0.075), Social Function subscale (*P *= 0.243), and Mental Health subscale (*P *= 0.716) (data not shown). In the responder analysis at week 24, a greater percentage of the adalimumab-treated group demonstrated changes exceeding the *a priori*-stated MCIDs compared with the placebo group for the SF-36 PCS (*P *< 0.001), BASFI (*P *< 0.001), and ASQOL (*P *< 0.001; Table 3). Sixty-seven percent of the adalimumab-treated patients exceeded the three-point MCID for the SF-36 PCS, compared with 39.8% of the placebo-treated patients.

**Table 3:** Responder status for primary PRO measures from baseline to week 24 of the ATLAS study

	Placebo (n = 107) n (%)	Adalimumab 40 mg every other week (n = 208) n (%)	*P *value^a^
SF-36 PCS, 0–50			
Responder, ≥ 3-point reduction	41 (39.8)	138 (67.0)	< 0.001
BASFI, 0–10			
Responder, ≥ 21% reduction	38 (35.5)	142 (68.6)	< 0.001
ASQOL, 0–18			
Responder, ≥ 1.8-point reduction	46 (43.0)	135 (64.9)	< 0.001

^a^*P-*values based on chi-square test.

ASQOL = AS Quality of Life Questionnaire; ATLAS = Adalimumab Trial Evaluating Long-Term Efficacy and Safety in AS; BASFI = Bath AS Functional Index; PCS = Physical Component Summary; PRO = patient-reported outcome; SF-36 = Short Form-36 Health Survey.

### Three-year open-label results

**Figure 1:**
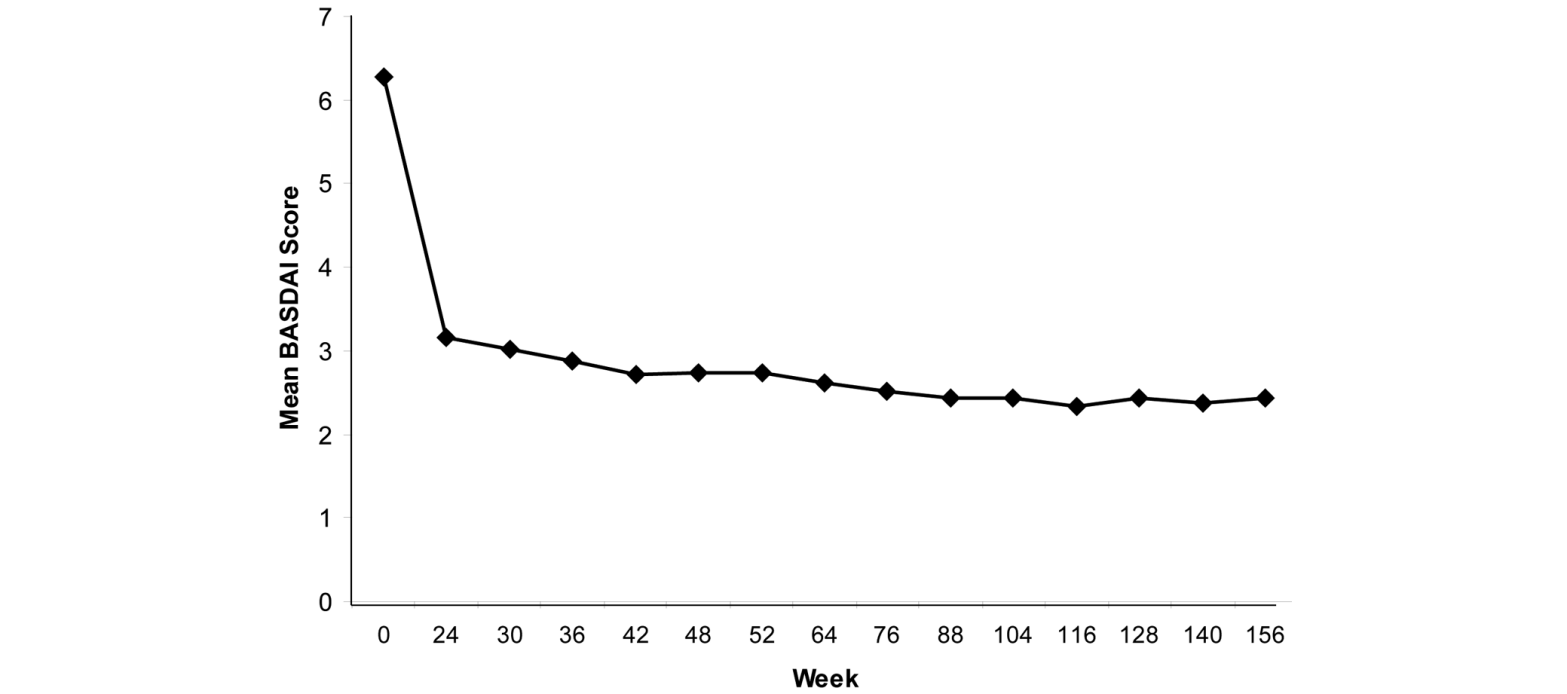
Mean BASDAI scores during long-term adalimumab treatment. Data are observed values for completers. Refer to Tables 4 or 6 for number of patients at each time point. The Bath Ankylosing Spondylitis Disease Activity Index (BASDAI) measures the severity of fatigue, spinal and peripheral joint pain, localized tenderness, and morning stiffness in patients with ankylosing spondylitis. BASDAI scores range from 0 to 10, with lower scores indicating less severe disease activity.

**Figure 2:**
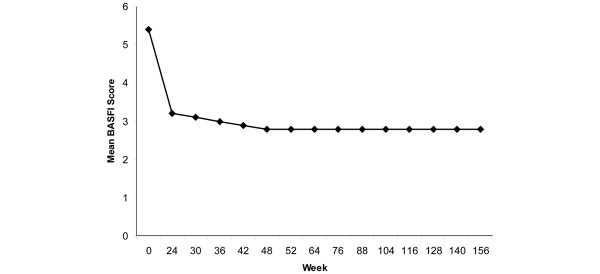
Mean BASFI scores during long-term adalimumab treatment. Data are observed values for completers. Refer to Tables 4, 5, or 6 for number of patients at each time point. The Bath Ankylosing Spondylitis Functional Index (BASFI) measures the degree of functional limitation in patients with ankylosing spondylitis. BASFI scores range from 0 to 10, with a lower score indicating less functional limitation.

Table 4 summarizes the changes from baseline to each follow-up assessment during the three-year treatment period for the BASDAI, BASFI, SF-36 PCS and MCS, SF-36 subscales, and ASQOL scores. Statistically significant changes from baseline were observed for all of the BASDAI (all *P *< 0.001) and BASFI scores (all *P *< 0.001), at all time points. The time course of mean scores for BASDAI and BASFI from baseline to the three-year endpoint is provided in Figures 1 and 2, respectively. Effect sizes for the BASFI scores ranged from 1.18 to 1.35. Based on LOCF, mean changes in BASDAI scores were -3.44 (*P *< 0.001) at year 1, -3.65 (*P *< 0.001) at year 2, and -3.49 (*P *< 0.001) at year 3. Mean change scores for BASFI via LOCF were -2.50 (*P *< 0.001) at year 1, -2.64 (*P *< 0.001) at year 2, and -2.63 (*P *< 0.001) at year 3.

**Table 4:** Change in mean PRO measures from baseline to week 24 and follow-up visits^a^

	Time point from baseline of the ATLAS study						
	
PRO measure	Week 24	Week 36	Week 52	Week 76	Week 104	Week 128	Week 156
BASDAI							
n	288	278	274	270	262	242	236
Mean ± SD change	-3.1 ± 2.42	-3.4 ± 2.41	-3.5 ± 2.55	-3.8 ± 2.33	-3.9 ± 2.44	-3.9 ± 2.39	-3.9 ± 3.39
BASFI							
n	288	^b^	274	270	261	242	236
Mean ± SD change	-2.2 ± 1.91	^b^	-2.6 ± 2.04	-2.8 ± 2.10	-2.9 ± 2.14	-2.9 ± 2.17	-3.0 ± 2.10
SF-36 PCS							
n	284	^b^	265	263	255	229	227
Mean ± SD change	8.2 ± 9.01	^b^	10.19 ± 9.50	10.8 ± 9.88	11.0 ± 9.88	11.3 ± 9.68	11.6 ± 9.65
SF-36 MCS							
n	284	^b^	265	263	255	229	227
Mean ± SD change	4.8 ± 10.27	^b^	5.6 ± 10.35	5.1 ± 11.06	5.7 ± 10.96	4.1 ± 10.84	5.6 ± 11.59
SF-36 Physical Function							
n	287	^b^	273	268	261	241	235
Mean ± SD change	15.6 ± 20.99	^b^	19.5 ± 21.38	21.8 ± 21.79	21.9 ± 22.26	22.2 ± 22.34	23.3 ± 21.94
SF-36 Role–Physical							
n	288	^b^	272	270	263	237	232
Mean ± SD change	30.3 ± 40.48	^b^	37.2 ± 40.81	35.2 ± 40.72	39.1 ± 41.75	36.1 ± 42.39	37.8 ± 43.90
SF-36 Bodily Pain							
n	288	^b^	274	270	263	237	233
Mean ± SD change	24.0 ± 21.78	^b^	29.0 ± 22.96	29.7 ± 23.24	29.9 ± 23.74	31.8 ± 24.42	31.7 ± 24.33
SF-36 General Health							
n	287	^b^	270	266	260	237	234
Mean ± SD change	8.7 ± 17.37	^b^	11.3 ± 18.68	12.7 ± 19.25	12.5 ± 19.92	12.0 ± 18.84	12.7 ± 18.71
SF-36 Vitality							
n	288	^b^	274	268	263	242	236
Mean ± SD change	16.3 ± 19.82	^b^	19.5 ± 19.96	20.1 ± 19.65	20.4 ± 20.05	18.4 ± 19.85	20.1 ± 19.70
SF-36 Social Function							
n	288	^b^	274	270	263	242	236
Mean ± SD change	16.1 ± 23.88	^b^	19.7 ± 24.50	18.5 ± 23.73	20.6 ± 25.36	17.9 ± 26.01	22.3 ± 25.16
SF-36 Role–Emotional							
n	286	^b^	271	268	260	235	230
Mean ± SD change	17.2 ± 41.24	^b^	21.0 ± 44.40	19.5 ± 46.74	21.5 ± 46.01	16.9 ± 44.80	20.0 ± 46.06
SF-36 Mental Health							
n	288	^b^	274	268	263	242	236
Mean ± SD change	8.8 ± 16.52	^b^	10.1 ± 17.15	10.2 ± 17.50	10.6 ± 16.63	8.7 ± 16.96	10.5 ± 18.32
ASQOL							
n	288	^b^	274	270	263	242	236
Mean ± SD change	-4.1 ± 4.23	^b^	-4.8 ± 4.41	-5.0 ± 4.32	-5.4 ± 4.28	-5.3 ± 4.35	-5.4 ± 4.36

^a^All values *P *< 0.001 compared with baseline based on paired Student *t*-test.

^b^Measure not assessed at this time point.

ASQOL = AS Quality of Life Questionnaire; ATLAS = Adalimumab Trial Evaluating Long-Term Efficacy and Safety in AS; BASDAI = Bath AS Disease Activity Index; BASFI = Bath AS Functional Index; MCS = Mental Component Summary; PCS = Physical Component Summary; PRO = patient-reported outcome; SD = standard deviation; SF-36 = Short Form-36 Health Survey.

**Figure 3:**
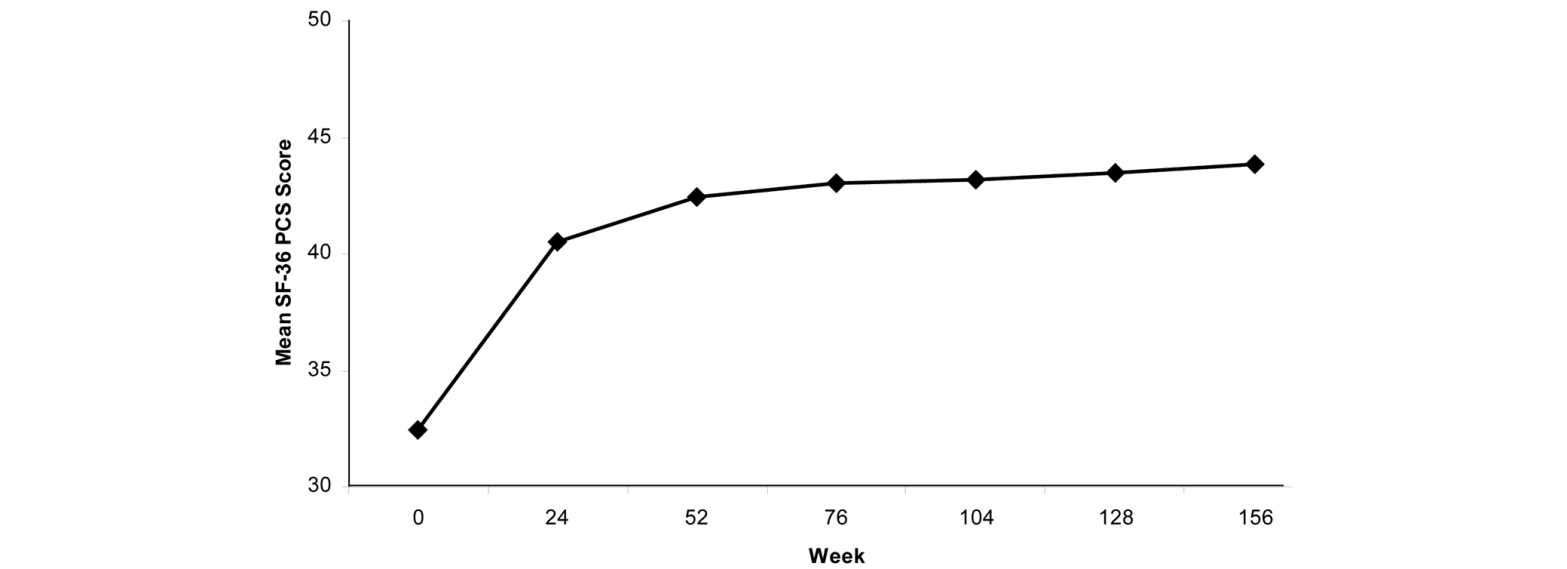
Mean SF-36 PCS scores during long-term adalimumab treatment. Data are observed values for completers. Refer to Tables 4, 5, or 6 for number of patients at each time point. The Short Form-36 Health Survey (SF-36) Physical Component Summary (PCS) is a composite score of four physical functioning domains of the SF-36: Physical Functioning, Role–Physical, Bodily Pain, and General Health. Greater scores indicate better health status.

In addition, statistically significant improvements were observed for both the SF-36 PCS and MCS scores (all *P *< 0.001). Mean PCS scores improved by 8.2 points at week 24 and by 11.6 points at year 3 (Figure 3). The effect sizes for the PCS scores were 0.87, 0.97, and 1.04 at years 1, 2, and 3, respectively. Mean changes in PCS scores by LOCF were 9.76 (*P *< 0.001) at year 1, 10.23 (*P *< 0.001) at year 2, and 10.03 (*P *< 0.001) at year 3. The SF-36 Physical Function score also demonstrated statistically significant improvements of 15.6 (week-24 assessment) to 23.3 points (after three years of adalimumab exposure). Comparable results were observed for the SF-36 MCS and other subscale scores (Table 4). Mean changes from baseline in ASQOL scores improved at all time points from week 24 through to year 3 (all *P *< 0.001). ASQOL effect sizes ranged from 1.11 to 1.26.

Table 5 summarizes the responder analyses for the SF-36 PCS, BASFI, and ASQOL for up to three years of adalimumab treatment. There were statistically significant percentages of responders for the SF-36 PCS scores from year 1 to year 3 (all *P *< 0.0001). The percentage of responders was sustained from 75.8% (year 1) to 81.9% (year 3). For the BASFI, we observed statistically significant percentages of responders (based on a 21% or greater improvement) across all assessments for up to three years of adalimumab treatment (all *P *< 0.001; Table 5). The responder rate was sustained from year 1 to year 3, ranging from 73.0% to 81.8%. Statistically significant percentages of ASQOL responders also were observed through up to three years of adalimumab treatment (all *P *< 0.001; Table 5). When a responder was defined as an improvement of at least 1.8 points, the percentage of responders was consistent from year 1 (77.0%) to year 3 (79.7%).

**Table 5:** Responder status for primary PRO measures from baseline to long-term follow-up visits

Primary PRO measure	Time point from baseline of the ATLAS study					
	
	Week 36	Week 52	Week 76	Week 104	Week 128	Week 156
SF-36 PCS responder, ≥ 3-point decrease from baseline						
n	^b^	265	263	255	229	227
Responders, n (%)	^b^	201 (75.8)^a^	197 (74.9)^a^	204 (80.0)^a^	182 (79.5)^a^	186 (81.9)^a^
BASFI responder, ≥ 21% reduction from baseline						
n	278	274	270	261	242	236
Responders, n (%)	203 (73.0)^a^	208 (75.9)^a^	211 (78.1)^a^	210 (80.5)^a^	195 (80.6)^a^	193 (81.8)^a^
ASQOL responder, ≥ 1.8-point reduction from baseline						
n	^b^	274	270	263	242	236
Responders, n (%)	^b^	211 (77.0)^a^	212 (78.5)^a^	213 (81.0)^a^	193 (79.8)^a^	188 (79.7)^a^

^a^*P *< 0.001; chi-square test for equal proportions was performed.

^b^Measure not assessed at this time point.

ASQOL = AS Quality of Life Questionnaire; ATLAS = Adalimumab Trial Evaluating Long-Term Efficacy and Safety in AS; BASFI = Bath AS Functional Index; PCS = Physical Component Summary; PRO = patient-reported outcome; SF-36 = Short Form-36 Health Survey.

The SF-36 Physical Function (all *P *< 0.001), Role–Physical (all *P *< 0.003), Bodily Pain (all *P *< 0.001), General Health (all *P *< 0.03), Vitality (*P *< 0.001), Social Function (all *P *< 0.001), and Role–Emotional (all *P *< 0.007) subscale scores also improved from baseline through year 3 (data not shown).

Table 6 summarizes the changes in selected PRO scores from the week-24 assessment to each open-label extension follow-up visit. For the BASDAI, small but statistically significant changes were observed for all assessments up to year 3 (all *P *< 0.009), except for that observed at week 30 (*P *= 0.240). Similarly, significant changes were observed from week 24 to each follow-up visit for BASFI scores (all *P *< 0.001), except for those observed at week 30 (*P *= 0.196) and week 36 (*P *= 0.100). These change scores suggest maintenance of improvement in disease activity and functional outcomes during up to three years of adalimumab treatment. SF-36 PCS scores also demonstrated statistically significant improvements from week 24 (*P *< 0.001; Table 6). Changes in PCS scores ranged from 1.8 (year 1) to 2.6 (year 3). No significant changes from week 24 were observed in the MCS (*P *> 0.05), with the exception of that observed at week 128 (*P *= 0.033). Statistically significant improvements in ASQOL from week 24 were observed through year 3 (all *P *< 0.002).

**Table 6:** Mean change in PRO measures from week 24 to each follow-up visit

PRO measure	Time point from baseline of the ATLAS study					
	
	Week 36	Week 52	Week 76	Week 104	Week 128	Week 156
BASDAI						
n	278	274	270	262	242	236
Mean change ± SD	-0.2 ± 1.41	-0.4 ± 1.59	-0.5 ± 1.65	-0.6 ± 1.87	-0.6 ± 1.80	-0.5 ± 2.88
*P*-value^a^	0.004	< 0.001	< 0.001	< 0.001	< 0.001	0.009
BASFI						
n	278	274	270	261	242	236
Mean change ± SD	-1.1 ± 10.68	-3.1 ± 13.07	-4.4 ± 14.22	-5.0 ± 15.90	-4.6 ± 15.87	-5.5 ± 14.27
*P-*value^a^	0.100	< 0.001	< 0.001	< 0.001	< 0.001	< 0.001
SF-36 PCS						
n	^b^	268	267	259	232	231
Mean change ± SD	^b^	1.8 ± 6.71	2.2 ± 7.35	2.4 ± 7.60	2.5 ± 8.54	2.6 ± 7.94
*P-*value^a^	^b^	< 0.001	< 0.001	< 0.001	< 0.001	< 0.001
SF-36 MCS						
n	^b^	268	267	259	232	231
Mean change ± SD	^b^	0.7 (7.55)	0.1 (7.50)	0.6 (7.37)	-1.2 (8.60)	0.2 (8.29)
*P-*value^a^	^b^	0.150	0.892	0.176	0.033	0.718
ASQOL						
n	^b^	274	270	263	242	236
Mean change ± SD	^b^	-0.5 (2.73)	-0.7 (2.78)	-1.0 (3.01)	-0.8 (3.43)	-1.0 (3.07)
*P-*value^a^	^b^	0.002	< 0.001	< 0.001	< 0.001	< 0.001

^a^*P-*values based on paired Student *t-*test.

^b^Measure not assessed at this time point.

ASQOL = AS Quality of Life Questionnaire; ATLAS = Adalimumab Trial Evaluating Long-Term Efficacy and Safety in AS; BASDAI = Bath AS Disease Activity Index; BASFI = Bath AS Functional Index; MCS = Mental Component Summary; PCS = Physical Component Summary; PRO = patient-reported outcome; SF-36 = Short Form-36 Health Survey.

## Discussion

This long-term, open-label extension of the ATLAS study demonstrated maintenance of improvement in physical function and HRQOL scores in patients with AS treated with adalimumab for up to three years. BASDAI scores demonstrated improvement in AS disease activity from baseline to the end of the 24-week double-blind treatment period and sustained improvement through to year 3. The benefit of adalimumab on physical function was demonstrated with sustained improvements in BASFI and SF-36 PCS endpoints over the three-year course of treatment. Improvements in the remaining SF-36 scores and the ASQOL provided further supportive evidence for more general and broad improvement in quality of life following therapy with adalimumab in patients with AS.

During the 24-week double-blind treatment period, analyses of the ATLAS study data [32] demonstrated greater improvements in the adalimumab-treated patients compared with the placebo-treated patients for changes in the BASDAI, BASFI, SF-36 PCS, and ASQOL scores. The SF-36 MCS scores did not differ between adalimumab and placebo groups. The SF-36 subscale scores, except for the Mental Health domain, also demonstrated results favorable for adalimumab compared with placebo. The changes seen in the SF-36 PCS scores exceeded the MCID and, therefore, are considered clinically meaningful. Of adalimumab-treated patients, 67% reported a clinically meaningful improvement in SF-36 PCS scores compared with 40% in the placebo group. The differences in the numbers of responders from baseline to week 24 for ASQOL scores also are considered clinically meaningful. Sixty-five percent of adalimumab-treated patients exhibited a clinically significant response on the ASQOL compared with 43% in the placebo group.

Over the course of the three-year open-label extension period, we observed significant improvements in all PRO scores, from baseline to each follow-up visit. Changes in the BASFI over time exceed the MCID for the BASFI (ie, ≥ 21% improvement) and indicate that the observed improvement over the first 24 weeks of the ATLAS study was maintained for up to three years of adalimumab treatment. Effect sizes for these changes in BASFI scores were large, ranging from 1.18 to 1.35. The BASDAI scores indicated similar consistent improvements from baseline over the course of the three-year open-label extension study. Seventy-six percent of study participants were classified as responders on the BASFI at year 1, and more than 80% were considered responders at years 2 and 3. These findings are consistent with other long-term follow-up studies of other TNF antagonists in patients with AS [8,11,25,30].

The changes observed in the SF-36 PCS scores over the three-year study demonstrated significant and clinically meaningful improvements in physical function and well-being with adalimumab treatment. Previous research has indicated that the MCID for the SF-36 PCS is 2.5 to 3.0 points [41]; the changes of 10.1 points at year 1 to 11.6 points at year 3 consistently exceeded this value and, therefore, are clinically meaningful. These improvements translate into effect sizes ranging from 1.26 to 1.45, which are considered very large for PRO endpoints [46]. By year 1, 76% of the participating patients were classified as responders on the PCS (improvement ≥ 3 points), and 80% and greater were classified as responders at years 2 and 3. These results are consistent with those of an earlier study of adalimumab in patients with AS, which demonstrated significant improvement in SF-36 PCS scores with 52-week open-label adalimumab treatment [47]. Studies of other TNF antagonists in patients with AS also have detected similar improvements in SF-36 PCS and MCS scores during long-term follow-up studies [8,11,22].

The results from the physical well-being–related SF-36 subscale scores (ie, Physical Function, Bodily Pain, and Role–Physical) provide additional supportive evidence for the SF-36 PCS findings. Changes in the Physical Function subscale score during long-term adalimumab treatment translate into effect sizes of 0.87, 0.97, and 1.04, for year 1, year 2, and year 3, respectively. Bodily Pain and Role–Physical subscale scores indicated consistent and robust changes from baseline over the course of the open-label extension. Improvements in Bodily Pain scores had large effect sizes (1.16 to 1.26), suggesting substantial improvements in self-reported pain during up to three years of adalimumab treatment (data not shown). Comparable degrees of improvement were also observed for the remaining SF-36 subscale scores, although relatively less change and effect sizes were observed for the Mental Health related scores.

Adalimumab treatment for up to three years improved AS-specific HRQOL, as measured by the ASQOL. Clear and consistent improvements in mean ASQOL scores from baseline were observed over the course of the open-label extension study. The observed effects are considered clinically significant as they exceed the 1.8-point MCID, and are associated with effect sizes ranging from 1.11 to 1.26. More importantly, 77% of patients in the open-label extension study were classified as responders at year 1, and 80% were responders at years 2 and 3.

There are several potential limitations associated with this study. First, the long-term results are based on an open-label extension study in which some placebo-treated patients switched to early escape adalimumab treatment from weeks 12 to 20, and all patients were switched to open-label adalimumab treatment after week 24. The addition of these patients to the long-term follow-up may have attenuated the observed effect of adalimumab on PRO endpoints. However, the data analyses found that despite the addition of these placebo-treated patients, the overall results suggest consistent and robust improvements in almost all PRO measures. Second, the physical function and HRQOL measures are based on patients' self-reports, and responses to the questionnaires may have been affected by the patients' knowledge that they were receiving adalimumab treatment. Although this knowledge may have had an initial impact on inflating PRO scores, it seems unlikely that this potential bias would have been sustained during the three-year follow-up period. Third, although 82% of patients participating in the open-label extension study provided complete assessments at year 3, study dropouts may have slighted inflated the PRO endpoint scores. And, finally, these data analyses were based on observed data. However, when we employed LOCF, the results did not differ substantively from the observed-analysis results. As expected, mean changes in PRO scores observed via LOCF were slightly lower than the mean changes seen with the observed analyses.

## Conclusions

In conclusion, the results of this open-label extension study demonstrated the sustained benefit of the statistically significant physical function and HRQOL improvements observed with adalimumab during the initial 24 weeks of double-blind treatment [16,32]. Consistent and robust effects were seen on BASFI and SF-36 PCS scores, both indicating impact on physical functioning and well-being for up to three years. These effects were also clinically meaningful. The remaining HRQOL outcomes, including the remaining SF-36 subscale scores and the ASQOL, extend and further support these findings. This study demonstrates that long-term treatment with adalimumab is associated with maintenance of effectiveness on physical functioning and well-being, as well as quality of life.

## Abbreviations

ANCOVA: analysis of covariance; AS: ankylosing spondylitis; ASAS20: Assessment in AS International Group Criteria for 20% improvement; ASQOL: AS Quality of Life Questionnaire; ATLAS: Adalimumab Trial Evaluating Long-Term Efficacy and Safety in AS; BASDAI: Bath AS Disease Activity Index; BASFI: Bath AS Functional Index; HRQOL: health-related quality-of-life; LOCF: last-observation-carried-forward; MCID: minimum clinically important difference; MCS: Mental Component Summary; NSAID: nonsteroidal anti-inflammatory drug; PCS: Physical Component Summary; PRO: patient-reported outcome; SF-36: Short Form-36 Health Survey; TNF: tumor necrosis factor; VAS: visual analog scale.

## Competing interests

DvdH has received consulting fees, research grants, and/or speaking fees from Abbott Laboratories, Amgen, Aventis, Bristol Meyers Squibb, Centocor, Pfizer, Roche, Schering-Plough, UCB, and Wyeth. JS has received consulting fees, research grants, and/or speaking fees from Abbott Laboratories, Bristol-Meyers Squibb, Centocor, Pfizer, Roche, Schering-Plough, UCB, and Wyeth. DR, NH, and CT are employees of United BioSource Corporation, which was contracted by Abbott Laboratories to complete the analyses reported here. KLG, RLW, and HK are employees of Abbott Laboratories and own shares of Abbott stock.

## Authors' contributions

Drs van der Heijde, Sieper, Wong, and Kupper (with other academic experts and members of Abbott Laboratories) designed the original clinical trial. Drs van der Heijde and Sieper were members of the ATLAS Study Group who collected the clinical data. Dr Revicki, Ms Thompson, and Ms Harnam completed the analyses. Dr Revicki, Dr Gooch, and Ms Harnam drafted the manuscript. All authors reviewed and approved the final content of the submitted manuscript.
